# Improvement of Upper Extremity Deficit after Constraint-Induced Movement Therapy Combined with and without Preconditioning Stimulation Using Dual-hemisphere Transcranial Direct Current Stimulation and Peripheral Neuromuscular Stimulation in Chronic Stroke Patients: A Pilot Randomized Controlled Trial

**DOI:** 10.3389/fneur.2017.00568

**Published:** 2017-10-30

**Authors:** Takashi Takebayashi, Kayoko Takahashi, Misa Moriwaki, Tomosaburo Sakamoto, Kazuhisa Domen

**Affiliations:** ^1^Graduate Course of Rehabilitation Science, Hyogo College of Medicine, Nishinomiya, Japan; ^2^Department of Occupational Therapy, School of Health Science and Social Welfare, Kibi International University, Takahashi, Japan; ^3^Department of Rehabilitation, School of Allied Health Science, Kitasato University, Sagamihara-shi, Japan; ^4^Department of Rehabilitation Medicine, Midorigaoka Hospital, Takatsuki, Japan; ^5^Department of Rehabilitation, Kansai Rehabilitation Hospital, Toyonaka, Japan; ^6^Department of Rehabilitation Medicine, Hyogo College of Medicine, Nishinomiya, Japan

**Keywords:** constraint-induced movement therapy, upper extremity, transcranial direct current stimulation, neuromuscular stimulation, stroke, rehabilitation

## Abstract

In this study, we investigated the effects of dual-hemisphere transcranial direct current stimulation (dual-tDCS) of both the affected (anodal tDCS) and non-affected (cathodal tDCS) primary motor cortex, combined with peripheral neuromuscular electrical stimulation (PNMES), on the effectiveness of constraint-induced movement therapy (CIMT) as a neurorehabilitation intervention in chronic stroke. We conducted a randomized controlled trial of feasibility, with a single blind assessor, with patients recruited from three outpatient clinics. Twenty chronic stroke patients were randomly allocated to the control group, receiving conventional CIMT, or the intervention group receiving dual-tDCS combined with PNMES before CIMT. Patients in the treatment group first underwent a 20-min period of dual-tDCS, followed immediately by PNMES, and subsequent CIMT for 2 h. Patients in the control group only received CIMT (with no pretreatment stimulation). All patients underwent two CIMT sessions, one in the morning and one in the afternoon, each lasting 2 h, for a total of 4 h of CIMT per day. Upper extremity function was assessed using the Fugl-Meyer Assessment (primary outcome), as well as the amount of use (AOU) and quality of movement (QOM) scores, obtained *via* the Motor Activity Log (secondary outcome). Nineteen patients completed the study, with one patient withdrawing after allocation. Compared to the control group, the treatment improvement in upper extremity function and AOU was significantly greater in the treatment than control group (change in upper extremity score, 9.20 ± 4.64 versus 4.56 ± 2.60, respectively, *P* < 0.01, η^2^ = 0.43; change in AOU score, 1.10 ± 0.65 versus 0.62 ± 0.85, respectively, *P* = 0.02, η^2^ = 0.52). There was no significant effect of the intervention on the QOM between the intervention and control groups (change in QOM score, 1.00 ± 0.62 versus 0.71 ± 0.72, respectively, *P* = 0.07, η^2^ = 0.43; treatment versus control). Our findings suggest a novel pretreatment stimulation strategy based on dual-tDCS and PNMES may enhance the therapeutic benefit of CIMT.

## Introduction

Approximately 15–30% of stroke survivors experience long-lasting upper extremity hemiparesis (1), with poststroke motor deficits of the upper extremity being a serious clinical concern. Therefore, treatments for upper extremity motor deficits are a critical component of stroke rehabilitation. In the 1980s, Taub et al. developed constraint-induced movement therapy (CIMT) as an intensive treatment for upper extremity motor deficit in chronic stroke patients (2). CIMT consists of task-oriented training for the affected upper extremity and a “transfer package” representing a behavioral method for enhancing adherence to treatment. Many previous studies have confirmed the effectiveness of CIMT for improving upper extremity function in chronic stroke patients (2–4). CIMT has been recommended by several guidelines for the improvement of the affected upper extremity function in chronic stroke patients (5, 6). In addition, several researchers have suggested that CIMT promotes plastic changes in the cortex, both contralateral and ipsilateral to the stroke lesion, in animal models of stroke and human stroke patients (7–9). The plasticity of the primary motor cortex is particularly important for the improvement in upper extremity motor function.

On the other hand, non-invasive brain stimulation has recently been shown to promote plastic changes when combined with standard physical or occupational therapy. Particularly, transcranial direct current stimulation (tDCS) has been used for priming cortical excitability during motor and behavioral training. The therapeutic mechanisms underlying improvement with tDCS involves effects on the activity of the Na^+^/Ca^++^ channel and *N*-methyl-d-aspartate receptor (10, 11), and promoting motor cortex plasticity, in a dose dependent, *via* activation effects on brain-derived neurotrophic factor and tropomyosin receptor kinase B (12). Cortex stimulation *via* tDCS is achieved using fine direct current from two electrodes placed on the scalp, which enhances the long-lasting modulation of cortical excitability through the depolarization or hyperpolarization of cells (13).

Two different tDCS strategies are used, namely anodal and cathodal stimulation, which increase or decrease the excitability level of cells, respectively. In an animal model, anodal tDCS of the affected hemisphere increases the excitability of the affected motor cortex for a few hours poststimulation (14). In healthy human subjects and in stroke patients, anodal tDCS of the affected hemisphere, combined with rehabilitation treatment, induced a modulation of cortical excitability of the affected motor cortex and promoted improvement of motor function on the affected side (15, 16). On the contrary, cathodal tDCS of the unaffected hemisphere combined with rehabilitation treatment decreased the excitability of the unaffected motor cortex, downregulating interhemispheric inhibition from the unaffected to the affected hemisphere, and improving motor function on the affected side in stroke patients (17). Recently, several researchers have recommended that both anodal and cathodal tDCS can be applied at the same time to act upon the affected and unaffected motor cortex, respectively (18–20). This tDCS strategy, termed dual-hemisphere tDCS (dual-tDCS), might further downregulate interhemispheric inhibition from the affected to the unaffected hemisphere. In addition, dual-tDCS was found to modulate intracortical and/or interhemispheric processing of primary motor cortex stimuli (21). In fact, Bolognini et al. (22) suggested that, compared to standard CIMT, dual-tDCS combined with CIMT yielded greater improvement in motor function of the affected upper extremity in chronic stroke patients.

While the current body of literature provides evidence that tDCS may promote rehabilitation-induced improvement in the motor function of the affected upper extremity in chronic stroke patients, several weaknesses of tDCS have been described. Uy and Ridding (23) suggested that, while anodal tDCS can increase the excitability of the cortex, the effects lasts <15 min. Nevertheless, these researchers also proposed that peripheral nerve stimulation (PNS) was a good strategy to prolong the effect of tDCS. The mechanism underlying the improvement in motor function with PNS are likely to be influenced by activity of the *N*-methyl-d-aspartate receptors (24–26) and GABAergic interneurons in the sensorimotor cortex. In fact, several researchers have demonstrated the added therapeutic effectiveness of combining tDCS, PNS, or peripheral neuromuscular electrical stimulation (PNMES), with rehabilitation (27–29). However, there are few articles that have shown the effectiveness of tDCS or dual tDCS combined with PNS or PNMES. Therefore, clear evidence of the effectiveness of this combined stimulation has not been established. Additionally, in CIMT’s study, the safety and effectiveness of this combined therapy (dual-tDCS combined with PNMES before CIMT) compared to CIMT alone, have yet to be examined.

Therefore, further research based upon the knowledge and experience obtained from previous studies is needed to develop a more efficient treatment strategy in stroke patients with motor deficit due to hemiparesis. In the present study, we first explored the hypothesis that the combination of dual-tDCS and PNMES, with adjustment of the level of muscle action potential evoked, would promote the effect of behavioral and motor therapy compared to conventional CIMT alone in chronic stroke patients with a paretic upper extremity. Additionally, we also evaluated the safety of the preconditioning treatment before CIMT compared to conventional CIMT alone.

## Materials and Methods

### Study Design

This pilot, multicenter, randomized, controlled study was carried out in accordance with the recommendations of the “Ethical guidelines for medical and health research involving human subject, Ministry of Education, Culture, Sports, Science, and Technology in Japan,” with written informed consent from all subjects. All study protocols were approved by the institutional review boards of each participating facility. The study was registered with the University Hospital Medical Information Network Clinical Trial Registry (UMIN000020927), which is a public trial registry.

### Subjects

Patients were recruited from outpatient stroke clinics affiliated with three participating facilities. The inclusion criteria were as follows: age, 20–90 years; and with a first stroke in chronic stage (>180 days from stroke onset). The exclusion criteria were as follows: bilateral or brain stem infarct or hemorrhage; voluntary extension of the metacarpophalangeal and interphalangeal joints of three or more fingers ≤10° or voluntary wrist extension ≤20°; severe impairment in balance or walking, indicated by the need for assistance for standing, walking or using the toilet; substantial use of the affected upper extremity before the intervention, indicated by a score of >2.5 points on the amount of use (AOU) scale of the Motor Activity Log (MAL); clear signs of dementia or cognitive disorder, indicated by a score <24 points in the Mini-Mental State Examination; severe aphasia or apraxia, preventing the patient from participating in the activities involved in the study intervention; presence of another uncontrolled medical condition or severe end-stage disease; and severe contraction in the area of the shoulder, elbow, wrist, or fingers.

### Sample Size Calculation

Before initiating the trial, we calculated that ten patients with chronic stroke should be enrolled in each group. A previous study suggested that a pilot study sample size should be 10% of sample size of project study (30). The largest CIMT study conducted to date was the EXCITE study (3), which enrolled about 100 patients in each group. Additionally, Hwetzog (31) recommended that pilot studies include 10–40 patients per group to provide an accurate estimation of treatment outcome. Therefore, we estimated 10 patients in each group would be sufficient.

### Randomization and Blinding

Participants were randomized into the control group (conventional CIMT) or the treatment group (dual-tDCS and PNMES plus CIMT) by a researcher blinded to group allocation and who was not directly involved in this study. Randomization was performed according to Zelen’s method, combined with minimization algorithm to control for the following factors: age (in years), time from stroke onset (in days), and baseline upper extremity score based on Fugl-Meyer Assessment (FMA) (32).

### Protocol for This Study

**Figure 1:**
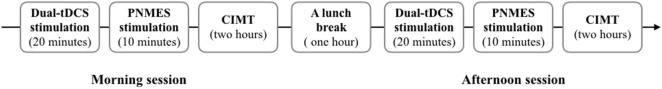
Daily rehabilitation protocol in the treatment group. Dual-tDCS, dual-hemisphere transcranial direct current stimulation; PNMES, peripheral neuromuscular electrical stimulation; CIMT, constraint-induced movement therapy.

The patients in the treatment group first received dual-tDCS for 20 min, followed immediately by PNMES, and then CIMT for 2 h. Pretreatment stimulation (dual-tDCS and PNMES) was applied both before the morning CIMT session and before the afternoon CIMT session (Figure 1). Dual-tDCS was performed using a DC-STIMULATOR PLUS (neuroConn GmbH, Ilmenau, Germany). For dual-tDCS, the anode was placed over the affected primary motor cortex (point C3 or C4 according to the 10–20 system), while the cathode was placed over the unaffected primary motor cortex (point C4 or C3 according to the 10–20 system). The following stimulation protocol was used for tDCS: constant current of 1-mA intensity (15), applied for 20 min (33), followed by PNMES performed using a TORIO stimulation system (Ito Co. Ltd., Tokyo, Japan), with two self-adhesive electrodes placed on the extensor digitorum muscle. Trains of electrical stimulation (20 Hz, on/off duty cycle 150/150μs; pulse duration, 300 µs) were applied at 1 Hz for 10 min. Stimulation intensity was set at a level where each patient reported mild paresthesia, but no pain, and minimal visible muscle contractions were evoked (23). Patients in the control group did not receive any stimulation. Sham stimulation was not used because the PNMES device used in this study did not have a setting permitting sham stimulation.

All patients received 4 h of CIMT (2 h in the morning and 2 h in the afternoon) from Monday to Friday for 2 weeks (10 consecutive weekdays). CIMT was provided by trained occupational therapists, according to a CIMT protocol described in detail elsewhere (34). CIMT is based on three main principles, namely: repetitive task-oriented training (shaping and task practice); training to facilitate the transfer of functional gains achieved in the clinical setting to the activities of daily living in real life (“transfer-package” training); and restraint of the less affected upper extremity using a mitt.

### Functional Assessment

We assessed the motor function of the affected upper extremity and its use in real-world behaviors, before and immediately after CIMT, using the FMA for the upper extremity, primary outcome (32), and MAL [AOU and quality of movement (QOM)], as the secondary outcome, respectively (35). The FMA for the upper extremity consists of 33 items, each scored on a 3-point ordinal scale: 0 point, cannot perform the action; 1 point, can perform the action only partially; and 2 points, can perform the action fully. Thus, the maximum possible upper extremity score was 66 points. The AOU and QOM scores, components of the MAL, indicate how much and how well, respectively, the affected upper extremity is used during 14 activities of daily living. For each activity of daily living, the patient rates the extent of the activity performed and how well it can be performed using the affected upper extremity. MAL scoring uses a 6-point Likert scale, ranging from 0 (never used) to 5 (used as prior to stroke). The mean AOU score reflects the frequency of the activity, whereas the mean QOM score reflects how well the activity was performed. The maximum value for the AOU and QOM scores is 5 points. The FMA and MAL assessments were performed by blinded and trained occupational therapists who was not directly involved in the treatment or patient allocation to intervention groups.

### Statistical Analysis

All data were analyzed using JMP version 13.0 (SAS Institute, Cary, NC, USA). Between-group differences in baseline characteristics were assessed using Fisher’s exact test (categorical data) or unpaired t test (ordinal data). Treatment effectiveness between the treatment and control group, which was assessed by analysis of covariance (ANCOVA), which was used to control for the baseline FMA score, and the AOU and QOM score of MAL, respectively. Finally, differences in upper extremity motor function, between baseline and postintervention, were assessed within each group using paired *t*-test. In all statistical comparisons, a *P* < 0.05 was considered statistically significant. The effect size index η^2^ (ANCOVA) and Δ (paired *t*-test) were also calculated. Data were presented as the mean (SD).

## Results

**Figure 2:**
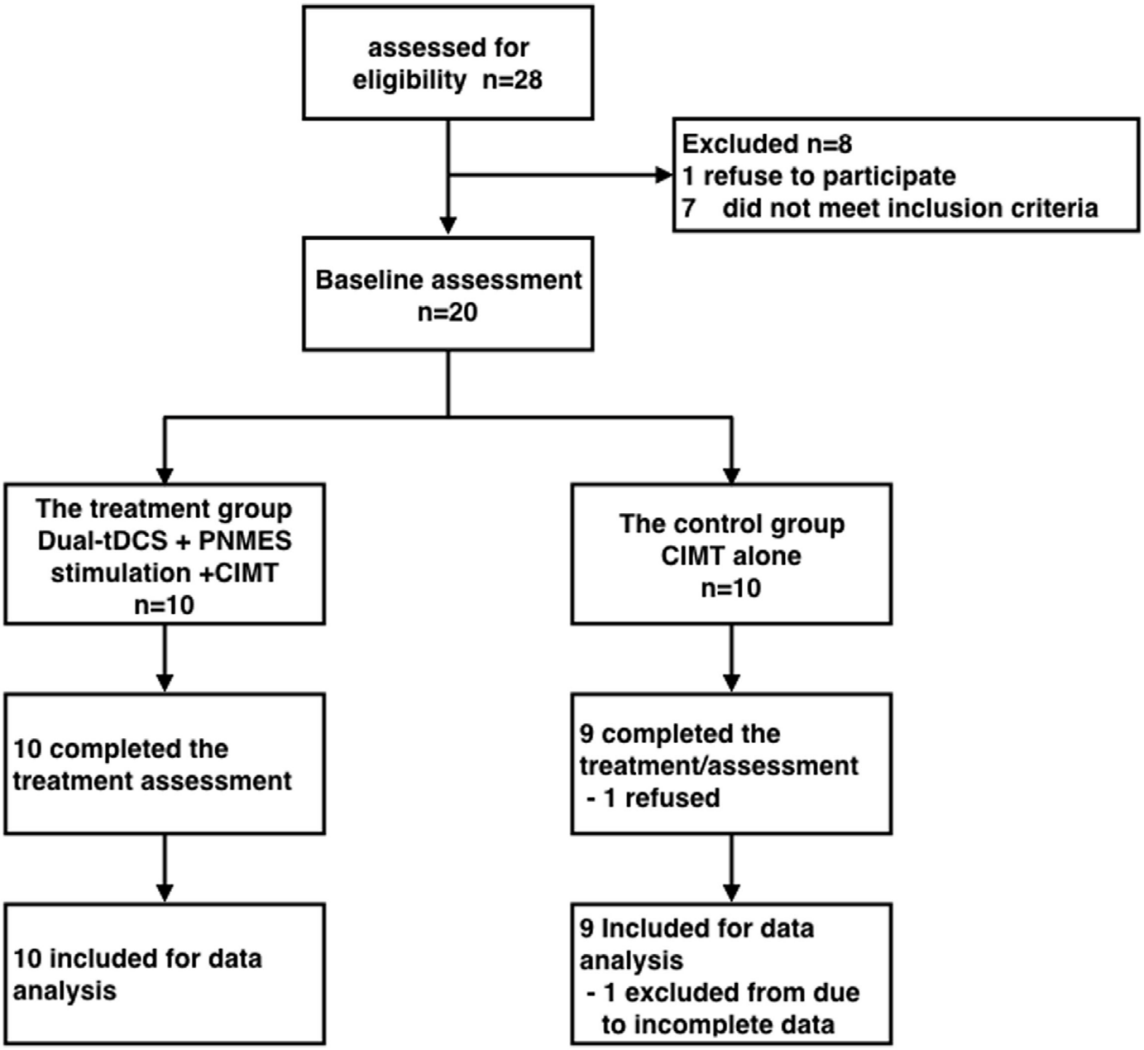
Flow-chart of patient enrollment in the study. Dual-tDCS, dual-hemisphere transcranial direct current stimulation; PNMES, peripheral neuromuscular electrical stimulation; CIMT, constraint-induced movement therapy.

A total of 28 candidates were screened between November 2014 and March 2017, of whom 20 patients were randomized into the treatment group or the control group (Figure 2). One patient allocated to the control group withdrew from the study after allocation. No adverse events were identified in either the treatment or control group. The two groups did not differ significantly in terms of baseline characteristics (Table 1).

**Table 1:** Baseline characteristics of the patients enrolled in the study.

Characteristic		Treatment group (*n* = 10)	Control group (*n* = 10)	Difference
				*P*-value
Age (years)		58.90 (8.28)	59.7 (15.82)	0.89
Gender, male/female		8/2	6/4	0.33
Time from stroke onset (days)		922.30 (693.95)	1,195.7 (1546.48)	0.62
Affected side, right/left		5/5	5/5	1.00
Hand dominance, right/left		10/0	9/1	0.30
Stroke type, hemorrhage/infarction		4/6	2/8	0.33
Site of lesion	Putamen	3	3	
	Prefrontal cortex	2	3	
	Corona radiata	1	1	
	Thalamus	3	2	
	Internal capsule	1	1	
Upper extremity score *via* FMA		43.00 (9.82)	44.00 (8.01)	0.81
AOU score *via* MAL		1.51 (0.78)	1.42 (0.79)	0.80
QOM score *via* MAL		1.55 (0.77)	1.36 (0.71)	0.57

*Data shown as average (SD) or number of observations*.

*AOU, amount of use; FMA, Fugl-Meyer Assessment; MAL, Motor Activity Log; QOM, quality of movement*.

The ANCOVA identified a greater improvement in the treatment than control group on the FMA upper extremity score [9.20 (4.64) versus 4.56 (2.60) for the treatment versus control, respectively; *P* < 0.01; η^2^ = 0.43] and MAL AOU score [1.10 (0.65) versus 0.62 (0.85), respectively; *P* = 0.02; η^2^ = 0.52]. However, no significant between-group difference in improvement was noted for the QOM scale of MAL [1.00 (0.62) versus 0.71 (0.72) for the treatment versus control, respectively; *P* = 0.07; η^2^ = 0.43] (Table 2).

**Table 2:** Outcomes of motor performance assessment at baseline and postintervention.

Outcome measure	Treatment group (*n* = 10)	Control group (*n* = 9)	Difference
			*P*-value^a^ (η^2^ value)^b^
**Upper extremity score *via* FMA**			
Baseline	43.00 (9.82)	45.44 (6.98)	
Postintervention	52.20 (8.28)	50.00 (8.82)	
Improvement	9.20 (4.64)	4.56 (2.60)	<0.01 (0.43)
**AOU score *via* MAL**			
Baseline	1.51 (0.78)	1.40 (0.83)	
Postintervention	2.61 (0.66)	2.02 (0.70)	
Improvement	1.10 (0.65)	0.61 (0.85)	0.02 (0.52)
**QOM score *via* MAL**			
Baseline	1.55 (0.77)	1.33 (0.74)	
Postintervention	2.55 (0.65)	2.04 (0.68)	
Improvement	1.10 (0.65)	0.71 (0.72)	0.07 (0.43)

*AOU, amount of use; FMA, Fugl-Meyer Assessment; MAL, Motor Activity Log; QOM quality of movement*.

*^a^Between-group comparisons involved analysis of covariance*.

*^b^Effect size was assessed using the η^2^ value*.

Within the treatment group, all motor performance indicators showed significant improvement from baseline: FMA upper extremity score, 43.00 (9.82) versus 52.20 (8.28), *P* < 0.01, Δ = 0.94, 95% confidence interval (95% CI) 5.89–13.22; AOU score of MAL, 1.51 (0.78) versus 2.61 (0.66), *P* < 0.01, Δ = 1.41, 95% CI = 0.62–1.66; QOM score of MAL, 1.55 (0.77) versus 2.55 (0.65), *P* < 0.01, Δ = 1.30, 95% CI = 0.52–1.53 (all values represent baseline versus posttreatment, respectively).

Similarly, within the control group, all indicators of motor performance improved significantly from baseline, namely: FMA upper extremity score, 45.44 (6.98) versus 50.00 (8.82), *P* < 0.01, Δ = 0.65, 95% CI = 2.91–6.49; AOU score of MAL, 1.40 (0.84) versus 2.02 (0.70), *P* = 0.04, Δ = 0.74, 95% CI = 0.05–1.20; QOM score of MAL, 1.33 (0.74) versus 2.04 (0.68), *P* < 0.01, Δ = 0.96, 95% CI = 0.23–1.21 (all values represent baseline versus posttreatment, respectively).

## Discussion

Our present findings indicate that, compared with patients who undergo behavioral and motor treatment alone, those who undergo behavioral and motor treatment after receiving *dual-tDCS* and PNMES recovered motor function (FMA upper extremity) and real-world (AOU in MAL) to a greater extent than patients who received conventional CIMT alone. Additionally, we confirmed that this combined treatment was as safe as the conventional CIMT alone.

### Effectiveness of the Dual-tDCS Combined with PNMES

Several researchers have defined the minimum clinically important difference regarding paretic upper extremity motor recovery in chronic stroke patients as an improvement above 4.25 points in the FMA upper extremity score and above 0.5 points in the AOU score (36, 37). In our study, both groups achieved improvements higher than these thresholds of clinically important difference on the FMA and AOU. Additionally, on the FMA upper extremity scale, the improvement was 4.64 points higher in the treatment than in the control group, and this between-group difference was also above the threshold for a clinically meaningful change. These findings suggest that behavioral motor treatment, combined with tDCS and PNMES, can provide meaningful improvement in upper limb function chronic stroke patients. On other hand, we found no significant between-group difference between in terms of the QOM score of the MAL. However, η^2^ of the effect size of the between-group difference was 0.43. According to Cohen, effect size assessed in terms of η^2^ is considered small effect for η^2^ < 0.01, medium effect for 0.01 < η^2^ < 0.06, and large effect for η^2^ > 0.14 (38). Therefore, although the *P*-value (*P* = 0.07) did not indicate a significant difference between the groups in terms of the QOM score of MAL, the η^2^ value indicated that this difference was indicative of a substantial effect of the combined treatment. This discrepancy might be explained by statistical errors (type II error or false negatives), most likely related to the small sample size and thus insufficient power in the statistical analysis. Additionally, Lang et al. (39) reported that the minimum clinically important difference regarding the improvement in the QOM score of MAL in stroke patients amounts to an increase of 1.00–1.10 points. In our study, the minimum clinically important difference in the QOM score of MAL was noted in treatment group but not in the control group, which supports the conclusion that there is consistently higher improvement in the treatment group even though the between-group difference in QOM score improvement was not statistically significant.

### Possibility of the PNMES in Neurorehabilitation Using the tDCS

The novelty of the present study lies in that we used a combination of dual-tDCS and PNMES, rather than only dual-tDCS or PNMES, to prolong the duration of the neural modulation effect. We found a clinically meaningful improvement in the motor function of the affected limb, reflected in the 9.2-point improvement in FMA upper extremity score [from 43.00 (9.82) to 52.20 (8.28)]. A previous study reported that dual-tDCS followed by CIMT achieved only a 6.3-point improvement in FMA upper extremity score (from 25.4 to 31.7 points) (22). This previous study used a similar CIMT treatment protocol as that applied in the present study (4 h per day for 10 consecutive weekdays). Compared to these previous observations, our findings indicated higher improvement in motor function on the affected side, although it should be noted that the degree of severity of deficit at baseline differed substantially between the two studies (patients in the present study were less affected upper extremity function compared to patients of the previous study). Therefore, our result showed that the novelty combination stimulation (dual-tDCS combined with the PNMES) strategy holds promise to improve the affected motor function rather than the dual-tDCS stimulation strategy alone. However, in this study, we could not make a strong claim about effectiveness of our novelty stimulation strategy, because we did not directly compare between the above two different stimulation strategies.

### Limitations and Scope for Further Study

The limitations of our study should be noted. First, the sample size of the present study was very small, with 10 patients in the treatment group and 9 patients in control group. We are planning to perform future trials after performing power calculations to estimate the minimum sample size necessary to ascertain the effectiveness of the combined treatment. The results obtained in this pilot study will serve for calculating the expected effect size. Second, we did not include a sham group because the device for PNMES did not have a setting for sham stimulation. Our result might include the placebo effect in which patients in the treatment group received some positive psychological effects because they understood that the PNMES might provide a positive treatment effect. Therefore, the placebo effect acting on the data regarding patients in the treatment group cannot be excluded. Third, in this study, we established the group that received CIMT alone as a control group. However, to investigate the effects of dual-tDCS combined with PNMES, we should establish a group that received CIMT combined with the dual-tDCS. We plan to address this issue in a further study.

### Conclusion and Clinical Implications

Despite the above limitations, the present trial clearly suggested that, compared to behavioral and motor rehabilitation alone, non-invasive stimulation with dual-tDCS and PNMES followed by behavioral and motor treatment provides greater effectiveness to enhance the recovery of motor function and real-world use of the affected upper extremity in patients with chronic stroke. Therefore, in chronic stroke patients, the novel pretreatment based on dual-tDCS and PNMES may enhance the therapeutic benefit of CIMT.

## Ethics Statement

This study was carried out in accordance with the recommendations of “Ethical guidelines for medical and health research involving human subject, Ministry of Education, Culture, Sports, Science, and Technology in Japan” with written informed consent from all subjects. All study protocols were approved by the institutional review boards of each participating facility (Hyogo College of Medicine, Kansai Rehabilitation Hospital, and Midorigaoka Hospital).

## Author Contributions

TT contributed equally in the study design, data collection, data analysis, and writing and reviewing the manuscript. KT, MM, TS, and KD contributed to writing and reviewing the manuscript.

## Conflict of Interest Statement

The authors declare that the research was conducted in the absence of any commercial or financial relationships that could be construed as a potential conflict of interest.
